# Towards standardised evaluative measurement of nature impacts: two spatial planning case studies for major Dutch lakes

**DOI:** 10.1007/s11356-014-2910-z

**Published:** 2014-04-27

**Authors:** P. J. T. M. van Puijenbroek, F. J. Sijtsma, F. G. Wortelboer, W. Ligtvoet, M. Maarse

**Affiliations:** 1 PBL Netherlands Environmental Assessment Agency, PO Box 303, 3720 AH Bilthoven, The Netherlands; 2 Faculteit Ruimtelijke Wetenschappen, Rijksuniversiteit Groningen, Landleven 1, 9747AD Groningen, The Netherlands; 3 Deltares, PO Box 85467, 3508 AL Utrecht, The Netherlands

**Keywords:** Lakes, Nature value, WFD, Nature 2000, IJsselmeer, Markermeer, Water birds

## Abstract

In the assessment of complex spatial planning projects, the ecological impacts and socio-economic impacts are fundamental to the evaluation. The measurements of ecological impacts of spatial plans have to be integrated in a standardised way. In the present paper, we analyse two Dutch case studies and apply the standardised Threat-Weighted Ecological Quality Area measurement. This measurement is developed to evaluate projects with terrestrial impacts but has not yet been applied for water evaluations. We aim to show how the use of a common measurement tool incorporates both ecological quality and degree of threat on criteria in the EU Water Framework Directive and Nature 2000. The measurements discussed here derive from two cases of cost–benefit analysis: The first case is the Markermeer, the second largest lake of The Netherlands, and a study on water quality improvement and nature restoration; an artificial island will also be the setting for a new residential area. The second case study is on water level management carried out on the IJsselmeer, the largest lake in the country. Results of our analysis show the potential impacts with a standardised method to the spatial distribution and quality of the ecosystems.

## Introduction

In most instances, spatial plans have to be evaluated for their impacts on nature quality and biodiversity. Many of the effects of spatial plans relate directly to the impacts and are therefore easy to determine. In other situations, however, one impact may have different effects on different locations in relation to the quality of the nature area. If there are several impacts or several different effects, the evaluation needs to integrate them in order to reach a final positive or negative effect.

In order to find the correct balance in the trade-off among (competing) goals and also evaluate the wide-ranging impacts of a project, a variety of evaluation tools can be used. Cost–benefit analysis (CBA) and variations of multi-criteria analysis (MCA) are the two most commonly employed tools capable of responding to this concern. Cost–benefit analysis takes as its starting point the preferences of individuals with regard to proposed changes (Boardman et al. 2011; Hanley and Barbier 2009; Mishan and Quah 2007; Pearce et al. 2006). MCA takes as its starting point the preferences of a decision-maker or group of decision-makers, or sometimes a broader group of stakeholders relevant to a project. As a project or policy decision will have various different impacts, MCA measures these impacts as separate criteria (Belton and Stewart 2002; Gamper and Turcanu 2007; Pomerol and Barba-Romero 2000). We have applied our approach to measure nature impacts in the framework of the MCCBA-approach to cost–benefit evaluation. This evaluation technique is a broad-based one, in which both CBA and MCA are combined in a standard and theoretically grounded way. A key characteristic of this approach is its use of standardised indices for recurring concerns in evaluation studies. For financial–economic impacts, MCCBA uses the discounted net-present value common to CBA. For health impacts, it uses the Quality (or Disability) Adjusted Life Years (Drummond et al. 2005; McPake et al. 2002; WHO 2009). For the evaluation of ecological impacts, the Threat-weighted Ecological Quality Area (T-EQA) index is applied (Sijtsma et al. 2011, 2013).

Many different evaluation systems have been defined for their quality of ecosystems (Brink 2000; EEA 2010a, b; Gregory et al. 2005; Jørgensen et al. 2013; Vačkář et al. 2012). But the T-EQA is designed in particular to standardise the measurement of biodiversity impacts. Biodiversity is the variety of life on earth within species, between species and across ecosystems. The most commonly used indicators of the method are the area of natural or semi-natural ecosystems and the numbers of species living within them. In the T-EQA, it is possible to measure the area of ecosystems as a natural unit (in hectares, or square kilometers) and then use species data to assess the quality of the area, which is known as Ecological Quality Area (EQA), the basis of our nature value indicator (Brink 2000; CBD 2007; Strijker et al. 2000). Ecological quality of terrestrial systems is calculated on the basis of the so-called mean species abundance (Brink 2000; Brink et al. 2002; MEA 2005). Every ecosystem is given a threat weight, thereby reflecting the degree of the risk to extinction or rare species to the system—at a specified spatial level. In this paper, the T-EQA measurement is used for the first time to evaluate changes in water-related biodiversity.

Several evaluation methods have been defined for biological quality in surface waters (Abbasi and Abbasi 2012; Jørgensen et al. 2013; Verdonschot 2012). As many indicators for biodiversity in terrestrial ecosystems are designed in response to threatened species (Bal et al. 2001; Vačkář et al. 2012), for aquatic systems, the indicators are based more generally on concentrations and abundances of organisms belonging to a trophic level of the ecosystem or a well-defined group of organisms (Jørgensen et al. 2013). However, for our purposes here, the most important indicator for the biological quality of surface water in The Netherlands is represented by the European Water Framework Directive (WFD) (EC 2000). The integrated biological quality refers to fish, aquatic invertebrates, algae and water plants. Indicators have been developed for each type of surface water (Evers et al. 2012; Molen et al. 2012).

Another biological quality system germane to our analysis are the Nature 2000 targets for the abundance of selected species (EC 1979, 1992). Quantified policy targets are defined for specific species and areas which can be used as a quantitative objective. As not all nature areas are Nature 2000, this method is useful only for quantified targets in designated Nature 2000 areas.

We discuss in this paper two spatial complex plans which have been evaluated on their effects on nature and biodiversity. The spatial plans involve the two largest lakes in The Netherlands, the IJsselmeer and Markermeer. The IJsselmeer area plan examines the increase in water level and fresh water supply in order to mitigate climate change. The spatial plan for the Markermeer includes both urban development and nature restoration. In both plans, a primary evaluation had to be carried out to account for the effects of the plans on Nature values. Both evaluations were part of a cost–benefit analysis, whereby biological effects had to be assessed together with economic effects, costs of measurements for nature restoration and the costs to elevate dikes (Bos et al. 2012; CPB/PBL 2009). However, note that the method provides a clear understanding of the physical ecological effects but does not provide the welfare effect of the ecological impacts. In these studies, the overall effects on nature and biodiversity were integrated into one quantified value so as to compare the different project alternatives of the spatial plans with each other.

In the next section, we will describe the two cases, Markermeer and Ijsselmeer, with their nature and policy targets on nature and water quality. Thereafter, we calculate the Nature values with the areas, their ecological quality and the corresponding weights with regard to different project alternatives. Results for the project alternatives are then presented in the form of Nature Points; advantages and disadvantages of the method are in the “Discussion,” and concluding remarks round out the paper.

## Material: the study area and spatial plans

**Fig. 1:**
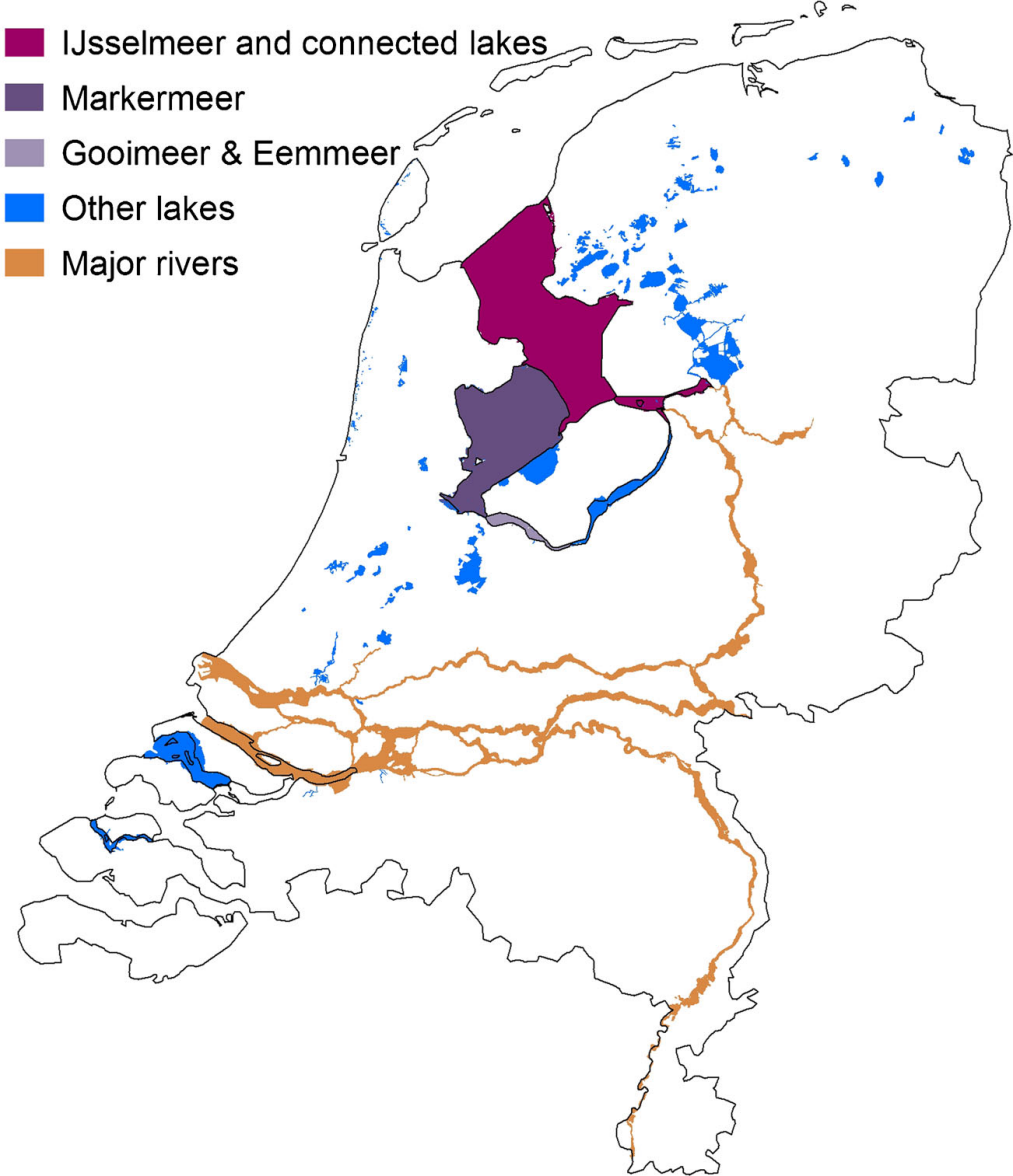
The IJsselmeer area and the Markermeer in The Netherlands

In our study here, we evaluate two integrated spatial plans and major decisions on water management and land use planning. The first case study is on the Markermeer and the connected lake IJmeer, which together comprise the second largest lake in The Netherlands with a surface area of 700 km^2^ (Fig. 1). The second case study concerns the IJsselmeer and connected lakes Ketelmeer, Vossemeer and Zwartemeer (together 1,200 km^2^). In this study, they are grouped together as the IJsselmeer area: the largest lake in The Netherlands. Both IJsselmeer and Markermeer have recently been reclaimed. The IJsselmeer was created by building the Afsluitdijk (completed in 1932), which enclosed the lake from the Waddenzee. Forty-seven years later, the Markermeer was formed by making the Houtribdijk (1979) which separated the IJsselmeer Lake from Markermeer.

### Case study one: housing and nature enhancement in the Markermeer

**Fig. 2:**
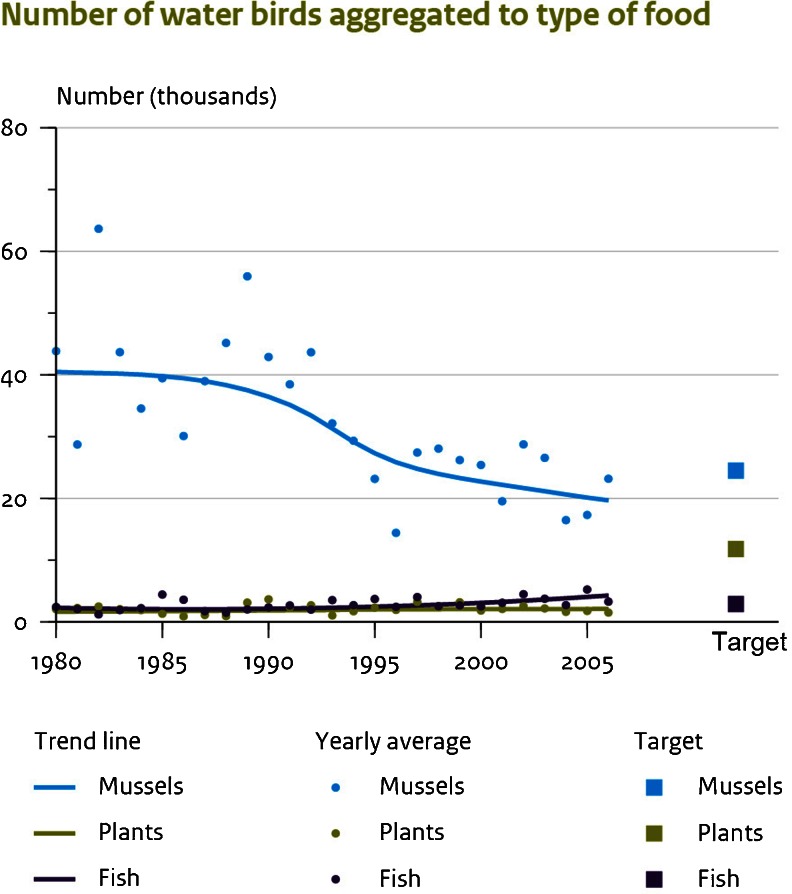
The number of birds foraging on Markermeer grouped into mussel-eating birds, plant-eating birds and fish-eating birds. They represent the Nature 2000 targets for the Markermeer and IJmeer

**Fig. 3:**
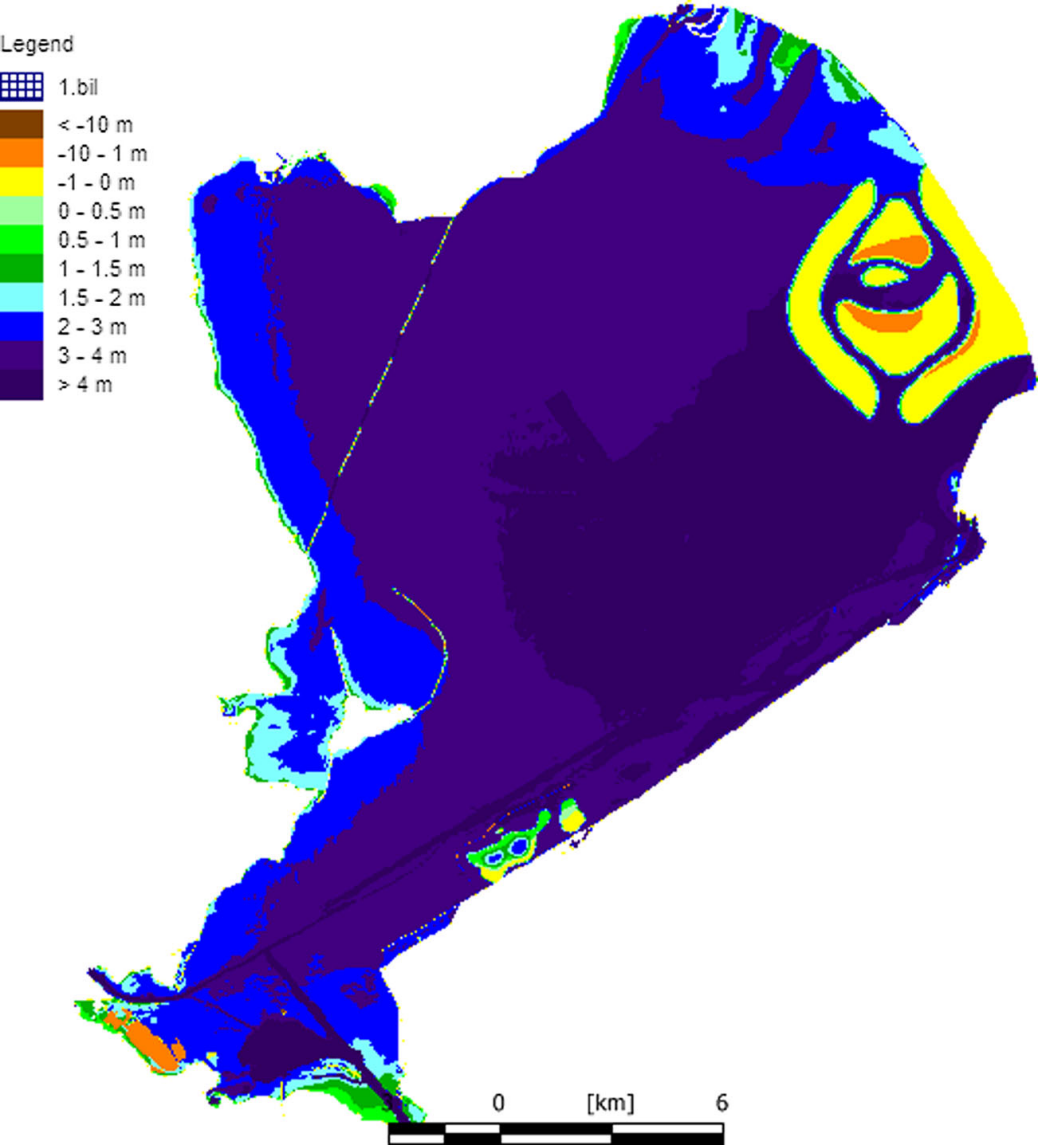
A schematic draft of the plans to improve nature quality in Markermeer

The Markermeer was transformed in 1930 from a sea to a fresh water lake, but one of the consequences of the work was that the silt sediment remains in suspension, thus resulting in a turbidity of 30 cm (Ministerie van Verkeer en Waterstaat 2008). This is a significant negative factor in relation to ecological quality. The total coast line is fortified with stones and water plants are scarce. The Markermeer is declining in its nature quality, as the number of mussel eating birds which feed on the lake is in decline (Fig. 2). However, given that these birds are part of the Nature 2000 target species (Programmadirectie Natura 2000 2009c), the policy decision was implemented which disallows negative effects to nature. In response, an integrated spatial plan for the Markermeer was drawn up (Samenwerkingsverband Toekomstagenda Markermeer–IJsselmeer 2009) to include (Fig. 3):

- an artificial area created in the south of the lake for residential building;
- an increase of recreation infrastructure on the south side of the lake;
- a large newly created wetland of 50 km^2^ in the north of the lake near the Houtribdijk;
- a partial enclosure of the north–west side of the lake (Hoornse Hop) to reduce sediment resuspension and promote the growth of water plants in the partly isolated part of the lake;
- a small shallow wetland protected from the waves by a small dike near Almere; and
- a deep pit in the centre of the lake to promote the deposition of suspended matter (and reduce turbidity).

The first two plans mentioned above have negative effects on the nature values. The artificial islands reduced the presence of mussels in the area. Negative effects were also recorded for other nature values, including an increase of disturbance for birds and bats. With the exception of the first two plans, all the other plans had some positive effects on nature quality. With the exception of the first two plans, all the other plans had some positive effects on. The aim of the plans overall was to improve the nature quality, restore the Nature 2000 targets of the lake and create a ‘surplus’ of nature quality in order to allow for future impacts. The total effect of all the positive and negative impacts had to be aggregated to a total effect on nature quality.

### Case study two: water level increase and freshwater reserve in IJsselmeer

The second study area is the IJsselmeer area, which has a fixed water level of 20 cm below mean sea level in summer and 30 cm below mean sea level in winter. The lake discharges to sea at low tide. An important function of the lake is that it serves as a reservoir to provide fresh water to a large part of the country during dry periods. When we examine possible future scenarios, in case of climate change and sea level rises, the lake will not be able to discharge to the sea under ‘normal’ situations. Therefore, in dry summers of some climate change scenarios, agriculture is expected to need more fresh water. To mitigate for climate change, in particular for fresh water needs and sea level rises, three project alternatives have been designed to change the water level of the lake in 2025, and 11 project alternatives have been drawn up for up to year 2100 (Bos et al. 2012). This great time span is required in order to achieve the investment required to pay for the major infrastructure in the event of sea level rises. In the present study, the present situation and the next three project alternatives are worked out (centimeters above or below mean sea level, the lowest level is only expected in incidentally dry years):

- Present situation: summer, −20 cm; winter, −30 cm; lowest level, −40 cm
- 80 cm increase: summer, +50 cm; winter ,−30 cm; lowest level, −40 cm
- 50 cm incidental decrease: summer, −10 cm; winter, −30 cm; lowest level, −80 cm
- 130 cm increase: summer, +110 cm; winter, +30; lowest, −40 cm

The major impact of sea level rise is expected to be a loss of terrestrial habitats beyond the dikes which would be flooded due to water level rise. These areas are particularly important for (breeding) birds; some islands are nesting places for thousands of terns, and other places are used by myriad flocks of geese in order to rest on the outer dikes. It is also expected that the distribution of aquatic habitats will change as the distribution of the depth zones changes; the depth of water has consequences for diving ducks which are not able to reach their food when water levels rise markedly. On the other hand, an incidental decrease of the water level can have a positive effect on the ecosystem for the growth of reed. In this study, the overall effects of the different water levels are calculated.

### Nature and water policies relevant to the lakes

Both the IJsselmeer and the Markermeer have been designated as Nature 2000 areas. The most important Nature 2000 targets (Table 1), however, are the water birds that feed on the lake or use the lake to rest, sleep or use as a stopover during migration (Programmadirectie Natura 2000 2009a, b, c, d). Other targets are specific habitats or certain species, such as the bat *Myotis dasycneme* that forages above the Markermeer, a vole, *Microtus oeconomus arenicola*, endemic to The Netherlands, and a small area of quaking bog on an island in the north west of the IJsselmeer. Also, the mussel, *Dreissena polymorpha*, is the most important food for birds in the lakes.

**Table 1:** The Nature 2000 targets for birds in the four lakes aggregated to breeding pairs, foraging and sleeping birds

		Species	Numbers
IJsselmeer	Pairs	10	12,438
	Forage	29	125,850
	Sleep	6	69,800
Zwarte meer	Pairs	5	343
	Forage	15	7,505
Ketelmeer en Vossemeer	Pairs	3	49
	Forage	17	9,386
Markermeer	Pairs	1	160
	Forage	15	46,000

In the scheme of the WFD, lakes are designated as water bodies, and their values are given in terms of water quality. The quality in accordance with the WFD is expressed as the ecological quality ratio (ekr) for the biological quality elements and provided in Table 2 (VenW et al. 2009). The target for the biological quality is a default 0.6, but, in this situation, for all biological targets and each water body, lower specific targets are also defined (Good Ecological Potential). To compare and evaluate the different water bodies, we have used the average biological quality of the four biological groups which represents the quality in respect to pristine situation.

**Table 2:** Biological quality of the lakes in the WFD (VenW et al. 2009)

	Phytoplankton	Macro benthos	Water plants	Fish	Average
IJsselmeer	0.35	0.38	0.17	0.61	0.38
Ketelmeer + Vossemeer	0.60	0.40	0.50	0.28	0.45
Zwartemeer	0.60	0.40	0.45	0.23	0.42
					0.41
Markermeer	0.45	0.41	0.53	0.54	0.48

## Methodology: calculate nature values

**Fig. 4:**
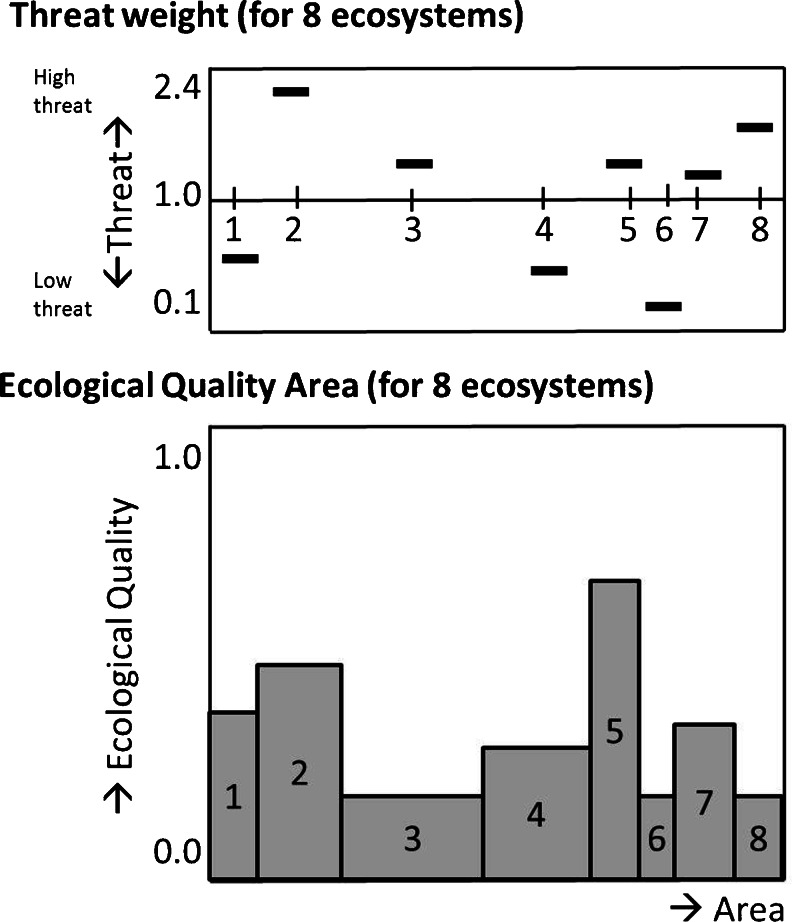
The elements of the T-EQA scores

Our next step is to calculate a T-EQA score using a general procedure shown in Fig. 4. First, the area of ecosystem relevant to the project under consideration is determined. Second, the local intactness/entirety/wholeness/robustness of the relevant ecosystem is calculated on the basis of the presence or abundance of characteristic species relative to the number or abundance that would be present in an intact ecosystem. This yields a score ranging from 0 to 1; we then multiply scores for the different ecosystems by their area which gives the EQA per ecosystem. The EQA score is thus reflected by the surfaces in lower part of Fig. 4. Finally, we multiply the EQA of the ecosystems with a standardised weight factor indicating the level of threat to the ecosystem; for instance, the relative number of red list species in an ecosystem may be used. The average weight of the eventual list of ecosystems on which the ecological evaluation data are based should be 1. Extremely threatened ecosystems should have the highest weight, while the most commonly occurring ecosystem with common species is expected to have the lowest weight. The multiplication factor between the highest and lowest weight is what defines the Threat weight at a given spatial scale. Quality for aquatic ecosystems is not defined by threatened species per se but rather by the food web characteristics of the system, therefore an alternative of the T-EQA for aquatic systems had to be defined.

The Threat-Ecological Quality Area is defined as:

$$ \mathrm{T}\hbox{-} \mathrm{EQA}={{\displaystyle \sum}}_{i=1}^n\left(\mathrm{Area}i\times \mathrm{Quality}i\times \mathrm{weightfactor}i\right); $$

where *i* represents different ecotopes and *n* is the number of identified ecotopes. The T-EQA is expressed in Nature Points. In order to calculate the T-EQA, the area, the quality and the weight factor of each ecotope must first be known. To evaluate the impacts of our case studies, we calculate and compare the starting T-EQA score with the scores from the different project alternatives.

### Area of ecotopes

**Fig. 5:**
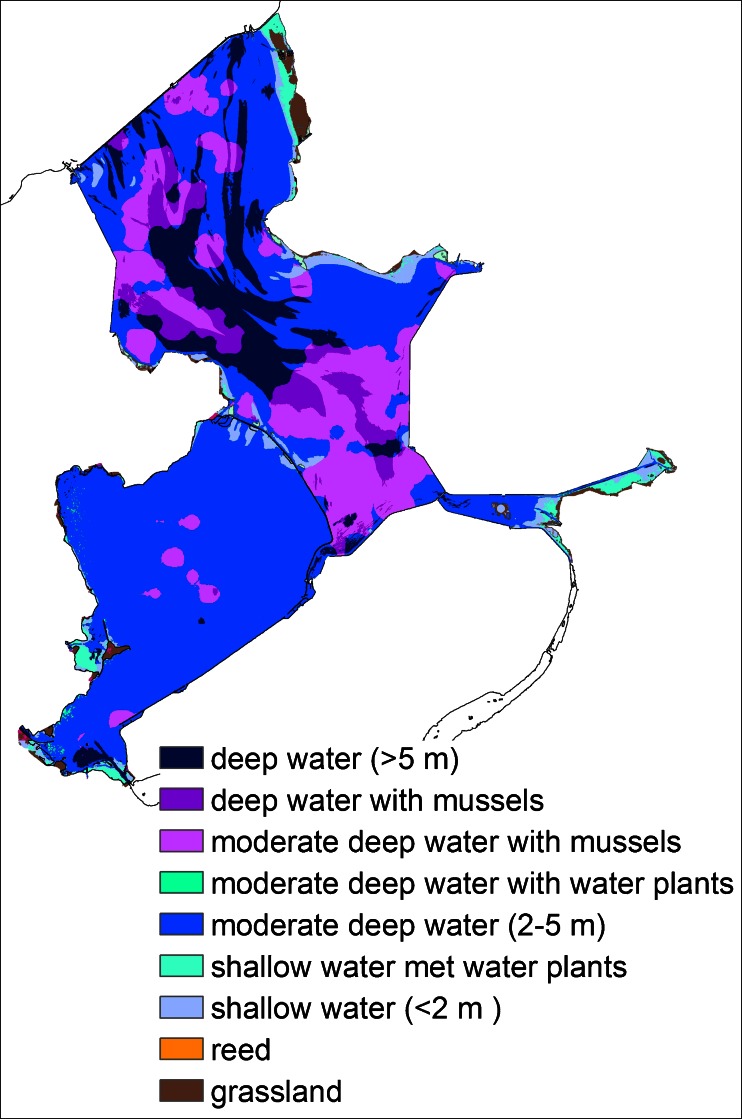
The spatial distribution of ecotopes in Markermeer and IJsselmeer area (Ecotopen map, RWS)

**Fig. 6:**
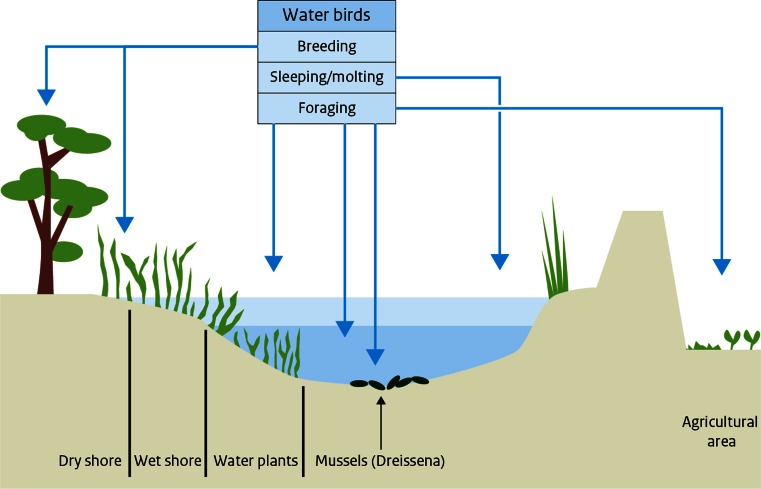
The different ecotopes in a lake with the ecological relation of birds in the ecosystem

To calculate the differences between the project alternatives, we made use of runs of the model Habitat for the project alternatives of the IJsselmeer area (Haasnoot and Wolfshaar 2009). This model calculated the area of ecotopes in the lake (Maarse and Noordhuis 2012). An ecotope is defined by Haasnoot and Wolfshaar (2009) as a homogeneous ecological unit, defined by abiotic (including but not limited to soil, climate, water availability and quality) and biotic factors (vegetation structure). In this case, the model differentiated among the ecotopes *Water with mussels*, *Water with water plants*, *Reed* and *Water with sandy soil*, and for each ecotope, the distributions between water depth zones were distinguished (Fig. 5). These ecotopes are characteristic for the most important ecological processes and for the abundant species of most birds (Fig. 6).

### Quality of ecotopes

The most important nature values are defined in Nature 2000 and WFD; together, they correspond to most of the biodiversity aspects. Biological quality within the WFD discussed above is used for the water quality of the lakes (Table 2). The results of the WFD for the lakes are comparable and are based on fish, macro benthos, algae and water plants. In so far as quality of ecotopes is concerned, it is calculated as the average standardised nature value of the biological groups. The WFD biological quality is restricted to the fresh water part of the area and is not developed for terrestrial areas. In the case of terrestrial areas, small ones are given the same quality as the rest of the lake, and only the new wetlands in Markermeer are given a higher quality.

### Threat weight factor for ecotopes in the case studies

The ecotopes of the lakes which have been identified have different relative importance within the total ecosystem. The shallow parts of the ecosystem have nature values for the benthic community and the surface water. In the deep parts of the lake, the majority of the biodiversity is in the open water, the pelagic part of the ecosystem, whereas the benthic system has less biodiversity. The nature restoration areas with terrestrial nature also have higher biodiversity than the deep parts of the lake. As we can see, various parts of the ecosystem have a different relative importance to the nature values of the system. To include the differences in ecosystems, weights for each ecotope were added; these weights are based on the type of bird group that feeds on the lake (Fig. 6). They are the top of the ecosystem trophic pyramid as consumers of fish, mussels and plants and thus integrate the lower parts of the food web (Gregory et al. 2005; Tomankova et al. 2012).

The food of birds is well known, so most bird species can be grouped into these ecotopes of the Habitat Model (Cramp et al. 1977; Nilsson 2005; Tomankova et al. 2012). The most important bird species which forage on mussels are the Coot (*Fulica atra*), Scaup (*Aythya marila*) and Tufted duck (*Aythya fuligula*); plant-eating birds are the Wigeon (*Anas penelope*), Mallard (*Anas platyrhynchos*) and Teal (*Anas crecca*). The most important fish-eating birds are the Cormorant (*Phalacrocorax carbo*), which breed in the neighbourhood and fish year round on the lake, Black tern (*Chlidonias niger*), present only a short time during the migration season and Common tern (*Sterna hirundo*), which breeds on an island in the IJsselmeer. The birds that dwell in reed are the Great reed warbler (*Acrocephalus arundinaceus*) and Sedge warbler (*Acrocephalus schoenobaenus*). Other bird species use the lake only for sleeping or resting during the migrating season, e.g. the Barnacle goose (*Branta leucopsis*), Golden plover (*Pluvialis apricaria*), Ruff (*Philomachus pugnax*) and White-fronted goose (*Anser albifrons*). A number of birds are omnivorous and eat mussels or plants, depending on the available food. In this case, the birds are grouped in their most favorite food for foraging on the lake and for the foraging depth.

Detailed quantitative information is available about the number of birds on both lakes (www.sovon.nl). The combination of number of birds, area and depth of ecotopes is combined to yield the number of birds per hectare (Table 3). Fish-eating birds are assumed to forage on the whole lake, independent of the depth of the lake and characteristic for the top pelagic species of the food web. The other weights are added to represent the biodiversity of the benthic and flora values. For these lakes, 95 % of the birds are also designated as Nature 2000 targets; it is therefore also used to compare with the threat-weighted factor for terrestrial nature quality.

**Table 3:** The weight factor for the ecotopes and differentiated to water depth

Water depth	Open water with benthic invertebrates	Open water with water plants	Open water (no benthic invertebrates or plants)	Reed, grass
>5	0.4	0.4	0.4	
4–5	0.4	0.4	0.4	
3–4	1.4	0.4	0.4	
2–3	2.0	0.4	0.4	
1–2	2.0	2.5	0.4	
0.2–1	2.0	1.9	0.4	
+0.2–0				2.3
>0.2				2.3

The weight factor is less for the Markermeer (0.2 instead of 0.4) for open water, as there are fewer fishing birds

### Project alternatives

Model runs from the Habitat Model for the lake IJsselmeer were available with the changes depicted in areas of ecotopes and corresponding water depths (Maarse and Noordhuis 2012). The water quality in the IJsselmeer is not supposed to change with these alternatives of water level change because most of the lake is deep water. A noteworthy effect of the alternatives with high water levels in the IJsselmeer is flooding of special islands that were constructed for birds to breed or rest. At present, thousands of common terns breed on the islands. Without reclaiming the island land, breeding would be impossible, as would rest and sleep. But these effects for rest and sleep are easy to compensate, and an alternative is available; therefore, these negative effects are ignored. On the other hand, the negative effect for breeding on the island is not compensated, and this is included as a reduction of the number of fish-eating birds: The weight factor for open water is reduced from 0.44 to 0.39. In other words, the highest trophic level for open water also depends on other factors than those specific to the lake.

In Markermeer, both positive effects to water quality and spatial changes in the area of ecotopes are expected. The creation of a new wetland occurs through a transformation of deep water to wetland with a consequent high nature quality (compared, for example, with the Oostvaardersplassen). The partial enclosure of the Hoornse Hop and the deep pits for sedimentation are presumed to have a positive effect on the lake quality, with the growth of more water plants and less turbidity in the entire lake. The newly created island for residential housing has a negative effect, as it has replaced the ecotope ‘water with mussels’ where many birds forage, with urban areas (without nature qualities). All changes in the plans were expressed in terms of a difference in area of ecotopes, or an increase in water quality of the lake.

## Results

### Results per project

**Fig. 7:**
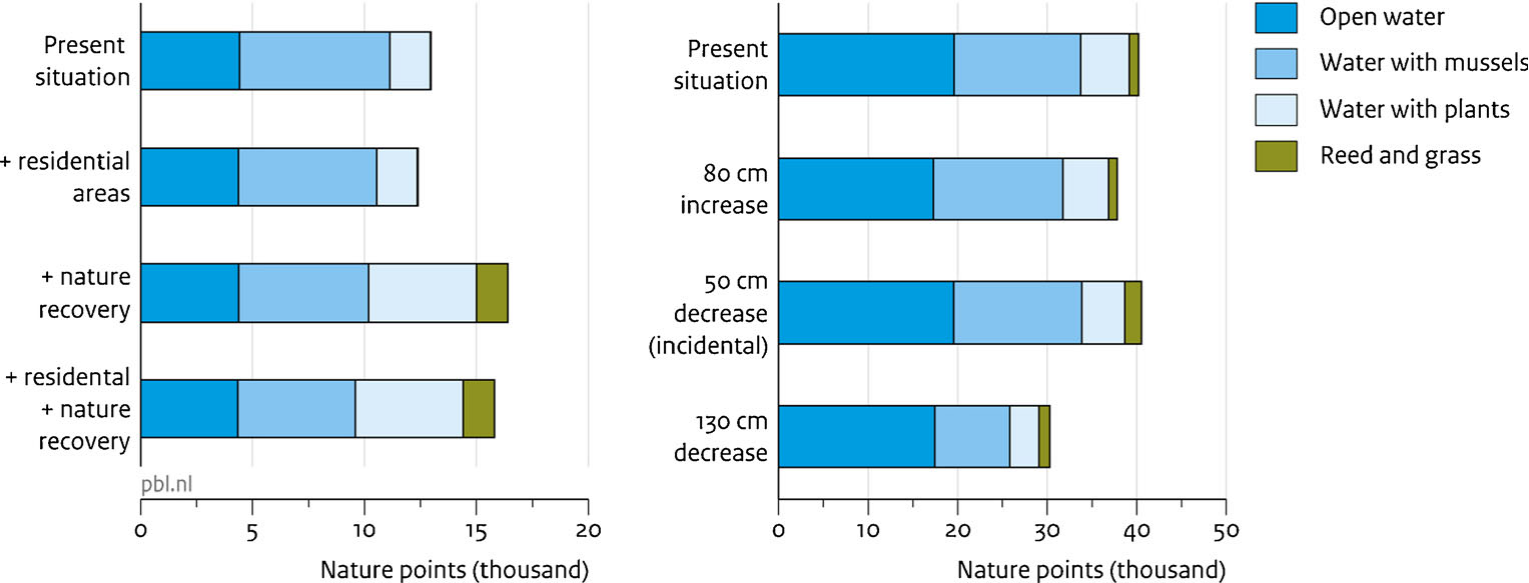
The results in Nature points for the Markermeer (*left*) and IJsselmeer area (*right*) for the present situation and three project alternatives

The results are expressed in Fig. 7 as ‘Nature points’ for the project alternatives of both lakes. The residential area in the newly constructed island in Markermeer had only a small negative effect on the nature values, as it reduced mussels in the area; in contrast, the artificial wetland incurred a major positive effect and thus compensated the loss of nature values over the last decades. The measurements to improve the turbidity also had a positive impact on the lake. The area with water plants will increase with the partial enclosure of the Hoornse Hop, compared with other small partly enclosed sections of the lake (Gouwzee). Water quality will also increase as a result of these measurements, affecting the whole lake by improving water quality. The total Nature points increased with the greater area of ‘water with plants’ and ‘reed’ of the wetlands.

In the IJsselmeer area, all project alternatives with water level rises had a negative effect on nature values. The project alternative with a 50 cm incidental decrease in the case of a dry summer had a slightly positive effect on the nature values, as it can have positive effects on the growth of reed in several places. The major part of the lake has moderately deep water, and changes in water level will have a negligent effect on the quality of the lake. The project alternative(s) with an increase of water level reduces the area of mussels which are presently available for diving ducks. When water is too deep, ducks cannot reach the mussels (Cramp et al. 1977). The areas of water plants are covered as a consequence of higher water levels during the spring season; with the turbidity of the water moreover, no light is available for the growth of plants. Flooding of the island reduces the number of birds feeding on the lake, therefore, the number of breeding birds diminishes. An increase of 130 cm of the maximum water level had a pronounced effect compared with an increase of 80 cm, as there is less ecotope ‘water with mussels’ in moderately deep water, with negative consequences for foraging birds.

### Comparison across projects

In this paper, we have shown the results of the separate case studies using the standardised T-EQA measurement. The T-EQA measure assists in decision making because different project alternatives can easily be compared. However, due to the standardisation, not only can alternatives now be compared within projects, but so too can comparisons be made across projects. In Table 4, we have added the total T-EQAs of the present situation in both lakes. Since they are weighted hectares, this is completely legitimate; different project alternatives of the different case studies can now be compared with each other. We have compared the five separate alternatives (excluding the combination of two in the Markermeer). Although the two case studies are completely separate initiatives, this may be helpful for overlooking the impacts of different policies and for assessing the size of the changes.

**Table 4:** Absolute nature value and changes in nature value for the project alternatives

	Present situation	Changes				
Both lakes		IJM +80 cm	IJM −50 cm	IJM +130 cm	MM housing	MM nature
Open water	24,019	−2,315	−30	−2,147	−45	−36
Water with mussels	20,814	328	164	−5,715	−544	−917
Water with water plants	7,271	−340	−618	−2,188	0	3,014
Reed and other land	1,065	−77	813	177	0	1,352
Total	53,170	−2403	328	−9,873	−588	3,413
Change of total		−5 %	1 %	−19 %	−1 %	6 %

Table 4 clearly shows that the incidental 50 cm dropping of the water level has a small positive impact, while housing in the Markermeer has a negative but also moderate impact (−1 %). We can observe that water level changes between 80 and 130 cm have severe effects: They reduce the ecological value of the combined lakes in the range of 5 % to 19 %. The Nature alternative is ambitious in its goal to enhance nature values in the Markermeer. It is a large-scale and complex initiative to realize, as we have seen above, among other things, a large ‘pristine swamp’. This initiative ‘only’ improves the nature quality by about 6 %. In making policy decisions, quantification helps in the interpretation and valuation of the trade-offs at stake. In this case, the +6 % of the ambitious Nature enhancing initiative seems to give the −19 % of the 130 cm change extra colour: Such a negative change is not easy to repair.

## Discussion

We are able to make several remarks on the method and results of this aggregated biodiversity indicator for presenting the effects of these spatial plans for large areas.

One concern about the use of this method is that only a selection of the present biodiversity is taken into account. Several bird species use the lake for resting or sleeping, and the majority of the species are designated as Nature 2000 targets (target of 69,000 geese for IJsselmeer). In this indicator, geese are not accounted for as regards the nature value of the lake; they are counted for the agriculture land because they feed on the agriculture land. Otherwise, we would encounter the problem of double counting, one for sleeping and one for foraging.

Specific Nature 2000 targets for species and habitats (the pond bat, the vole and certain habitats) are ignored in the Nature value calculation, as the effects of these species and habitats are difficult to predict.

Another noteworthy concern is the weight factor for the final results. In this case, the given weight is based on the group of foraging birds as the most important species of the highest level of the trophic pyramid (excluding human fishery and large adult predatory fish). This group of birds had a large overlap with the Nature 2000 species of the lakes. Therefore, the weight factor is comparable with that of terrestrial ecosystems (Sijtsma et al. 2011). The weights range between 0.2 (open water in Markermeer) and 3 (reed, water with plants or mussels); this is a factor 15 between the most important ecotope and less important ecotope. In other studies, a range in weight factors have a comparable range (Sijtsma et al. 2009; Wessels et al. 2011).

An important consideration is that many birds forage in the lake, but they breed elsewhere. In these lakes, there are two important species, the cormorant and the common tern. Both birds forage in the lake, but the cormorant breeds elsewhere, while the common tern breeds on the island in the lake. In this case, the cormorant is not affected by an increase of water level, but the common tern cannot breed on the islands with water levels over a certain depth. Therefore, the abundance of fish-eating birds depends on available food in the lake and also on the ability to breed in the neighbourhood of the lake. In this case, the weight factor depends on the availability of breeding places for birds.

Another aspect is that ecological effects are also more complex than a direct dose–response relation, which are not all included in this study. For example, a major change of the percentage of ‘water with plants’ could impose consequences for the fish community or the algae concentration in the lake. These effects are complex, and more research is needed to investigate them. In the current two cases, the situation is not expected to incur much change in the area of water with plants; therefore, no effects to other biological groups are expected. Moreover, the effects on the land–water interface are important for these project alternatives, but they are difficult to determine. Incidental low water level in dry summers in Ijsselmeer area is assumed to have positive effects on the growth of reed.

The T-EQA is calculated on the area, quality and weight factor for ecological quality for each ecotope. The applied quality parameter is taken from the WFD for biological quality. The biological quality of the WFD is based on monitoring data of locations in different ecotopes, but in the biological quality is this aggregated to a biological quality for the lake. It would be preferred if the biological quality was available for each ecotope for a better defined quality for the ecotopes.

The most important improvement of this assessment is its ability to access the WFD biological quality for each ecotope instead of for the whole lake. Terrestrial and aquatic ecosystems have different quality assessments, different scales and different targets. In this assessment, the two different systems had to be integrated. The weight factor is especially important for the differences ion biodiversity between terrestrial and aquatic systems. In combination with the previous improvements, the weight factor could also be improved. Research is underway to refine the weight factors for these assessments. Despite its drawbacks, the presented indicator is based on the most important groups of biodiversity and represents an approved model for calculating the area of ecotopes.

## Conclusion

In this study, an indicator has been developed and applied to two cases for the largest lakes in The Netherlands. This method includes the biological groups algae, water plants, macro benthos, fish and birds and integrated the results into one indicator. The indicator, T-EQA, has been calculated by multiplying the area, quality and weight factor for all available ecotopes. The quality is based on the average of the four biological groups in the WFD evaluation. The changes in the area of ecotopes have been calculated using the model Habitat. Weight factors are important in calculating the T-EQA as not all ecotopes have equal biodiversity values. The abundance of common species is more important in aquatic ecosystems, especially in the large lakes under consideration than the presence of rare species. Therefore, a weight factor for aquatic systems has been developed for the abundance of foraging species, as they represent the top of the trophic pyramid.

Through the use of the T-EQA method, the Nature values were presented at an early stage in the decision process on spatial development and water management. With the aggregation to one index, the nature values have been included in the decision. The results of the Markermeer and IJsselmeer area can be integrated because they have been calculated with the standardised method. However, with this approach, local differences are neglected; some groups, such as birds that use the lake to sleep, are not included. Further research is needed to ascertain the biological quality for specific ecotopes instead of a whole lake, in order to improve the weight factors for the relative importance of different ecosystems and to integrate both aquatic and terrestrial nature values.
